# Extracellular vesicle-Serpine-1 affects neural progenitor cell mitochondrial functions and synaptic density: modulation by amyloid beta and HIV-1

**DOI:** 10.21203/rs.3.rs-2551245/v1

**Published:** 2023-02-15

**Authors:** Ibolya E. András, Nelson Serrano, Irina Djuraskovic, Nikolai Fattakhov, Enze Sun, Michal Toborek

**Affiliations:** University of Miami Miller School of Medicine: University of Miami School of Medicine; University of Miami Miller School of Medicine: University of Miami School of Medicine; University of Miami Miller School of Medicine: University of Miami School of Medicine; University of Miami Miller School of Medicine: University of Miami School of Medicine; University of Miami Miller School of Medicine: University of Miami School of Medicine; University of Miami Miller School of Medicine: University of Miami School of Medicine

**Keywords:** Serpine-1, extracellular vesicles, blood-brain barrier, HIV-1, amyloid beta, neural progenitor cells

## Abstract

Brain endothelial extracellular vesicles carrying amyloid beta (EV-Aβ) can be transferred to neural progenitor cells (NPCs) leading to NPC dysfunction. However, the events involved in this EV-mediated Aβ pathology are unclear. EV-proteomics studies identified Serpine-1 (plasminogen activator inhibitor 1, PAI-1) as a major connecting “hub” on several protein-protein interaction maps. Serpine-1 was described as a key player in Aβ pathology and was linked to HIV-1 infection as well. Therefore, the aim of this work was to address the hypothesis that Serpine-1 can be transferred via EVs from brain endothelial cells to NPCs and contribute to NPC dysfunction. HBMEC concentrated and released Serpine-1 via EVs, the effect that was potentiated by HIV-1 and Aβ. EVs loaded with Serpine-1 were readily taken up by NPCs, and HIV-1 enhanced this event. Interestingly, a highly specific Serpine-1 inhibitor PAI039 increased EV-Aβ transfer to NPCs in the presence of HIV-1. PAI039 also partially blocked mitochondrial network morphology and mitochondrial function alterations in the recipient NPCs, which developed mainly after HIV + Aβ-EV transfer. PAI039 partly attenuated HIV-EV-mediated decreased synaptic protein levels in NPCs, while increased synaptic protein levels in NPC projections. These findings contribute to a better understanding of the complex mechanisms underlying EV-Serpine-1 related Aβ pathology in the context of HIV infection. They are relevant to HIV-1 associated neurocognitive disorders (HAND) in an effort to elucidate the mechanisms of neuropathology in HIV infection.

## Introduction

Extensive evidence indicates amyloid pathology in HIV-1 infection. Studies reported that HIV-infected brains have increased amyloid beta (Aβ) deposition mostly in the perivascular space [1–4] when compared to age-matched controls [1, 5–9]. These earlier findings have recently been reevaluated and expanded, as new evidence from autopsy of HIV-1 brains did not confirm an overall enhanced Aβ deposition. Instead, duration of infection was correlated with Aβ accumulation independent of age. These findings suggest that the length of HIV infection, and not the age of patients, predicts elevated brain Aβ levels, confirming an accelerated brain senescence in people living with HIV [10]. For example, methylome-wide analysis of chronic HIV-1 infection revealed five-year increase in biological age of infected individuals [11]. HIV-related age acceleration has been shown to be associated with reductions in total gray matter using epigenetic age as a biomarker for age acceleration [12]. Evidence indicates that increased and persistent senescence during HIV-1 infection contributes to chronic inflammation, immune failure and mitochondrial dysfunction [13–15].

It was demonstrated that the blood-brain barrier (BBB) is critical for Aβ homeostasis and contributes to elevated Aβ deposition in the brain [16, 17]. Aβ transport into the brain across the BBB involves BBB transfer mechanisms such as the receptor for advanced glycation end products (RAGE) [18]. Moreover, we found that HIV-1 contributes to Aβ accumulation in brain endothelial cells via upregulation of RAGE [19]. Extracellular vesicles (EVs) also appear to be important players in Aβ pathology by carrying biologically active proteins and genetic material. We demonstrated that exposure to HIV-1 increases EV release from human brain microvascular endothelial cells (HBMEC) and alter their Aβ cargo [20]. EVs having diverse origin, size, and cargo can be taken up by the target cells via several routes [21]. Importantly, HBMEC-derived EVs (HBMEC-EVs) carrying Aβ can be transferred to other cells of the neurovascular unit, including neural progenitor cells (NPCs), causing NPC dysfunction and their aberrant neurogenesis [22]. The mechanisms of this EV-mediated Aβ pathology are not clear; however, proteomics of HBMEC-EVs revealed a complex protein cargo with elaborate functional interactions as mapped in STRING [23]. These maps had several “hubs” with more connections to other proteins. One of the main proteins at such crossroad interactions was Serpine-1, known also as plasminogen activator inhibitor 1, PAI-1.

Serpine-1 is crucial for the coagulation/fibrinolysis homeostasis. By inhibiting tissue plasminogen activator (tPA) and urokinase type activator (uPA), Serpine-1 promotes coagulation [24]. These processes are important in both HIV-1 infection and Alzheimer’s amyloidopathy. For instance, endothelial dysfunction in HIV-1 infection is associated with leucocyte recruitment, platelet adhesion and aggregation, blood clotting activation and fibrinolysis derangement that may be responsible for occlusive thrombotic events [25]. In addition, HIV-1 replication alters the composition of extrinsic pathway coagulation factors and increase thrombin generation, leading to a procoagulant state [26]. HIV-1 infection constitutes a higher risk of cardiovascular events, including stroke and blood-brain barrier (BBB) disruption, due to coagulation abnormalities and dyslipidemia [27]. Regarding Aβ pathology, activation of plasmin via the coagulation cascade, results not only in fibrynolysis but also in degradation of APP and Aβ. Consequently, Serpine-1, by decreasing tPA-plasminogen-plasmin-mediated APP/Aβ degradation, can contribute to an increase in Aβ levels [28].

Because both HIV-1 infection [29] and Aβ pathology [30, 31] were linked to elevated levels of Serpine-1, the aim of the present study was to address the hypothesis that Serpine-1 can be transferred via EVs from brain endothelial cells to NPCs affecting their functions. Mechanistically, we focused on the role of EV-associated Serpine-1 (EV-Serpine-1) in the NPC mitochondrial changes in the context of HIV-1. We also evaluated the involvement of Serpine-1 in NPC synaptic protein alterations. The obtained results indicate that EV-Serpine-1 may be a crucial player in vascular Aβ pathology in HIV-infection.

## Materials And Methods

### Cell cultures

#### Human brain microvascular endothelial cells (HBMEC).

HBMEC used in the present study represent a stable, well characterized, and differentiated human brain endothelial cell line [32]. Briefly, normal human brain endothelial cells were transduced by lentiviral vectors incorporating human telomerase or SV40T antigen. Among several stable immortalized clones obtained by sequential limiting dilution cloning of the transduced cells, the hCMEC/D3 cell line (referred here as HBMEC) was selected as expressing normal endothelial markers and demonstrating blood–brain barrier characteristics. HBMEC for the present study were supplied by Dr. Couraud (Institut Cochin, Paris, France). HBMEC were cultured on collagen type I (BD Biosciences Pharmingen, San Jose, CA) coated dishes in EBM-2 medium (Clonetics, East Rutherford, NJ) supplemented with VEGF, IGF-1, EGF, basic FGF, hydrocortisone, ascorbate, gentamycin and 0.5% exosome depleted fetal bovine serum (Exo-FBS; System Biosciences, Mountain View, CA).

### Human neural progenitor cells (NPCs)

An immortalized NPC line ReNcell VM, derived from 10-week human ventral mesencephalon, was obtained from Millipore and cultured according to the manufacturer’s protocols. The cells were validated for high expression of Sox2 and nestin as well as for their self-renewal and differentiation capacity. Cells were grown on laminin coated tissue culture dishes in a maintenance medium (Millipore), containing 20 ng/ml FGF-2 and 20 ng/ml of rhEGF. Cells were used for experiments at < 80% confluence, three days after plating.

### HIV-1 infection and treatment factors

HIV-1 stock was generated using human embryonic kidney (HEK) 293T cells (ATCC) transfected with pYK-JRCSF plasmid containing full-length proviral DNA. Throughout the study, HBMEC were exposed to HIV-1 particles at the p24 level of 30 ng/ml as previously reported [22]. Treatment was terminated by removing cell culture media containing HIV-1, followed by washing the cells with PBS.

Aβ (1–40) and Aβ (1–40) HiLyte 647 were purchased from Anaspec (San Jose, CA) and dissolved in PBS. Freshly solubilized Aβ solutions without pre-aggregation were used for experiments as such a form of Aβ was demonstrated to induce proinflammatory reactions [33]. Aβ (1–40) HiLyte was dissolved first in a basic buffer (0.1 M NH_4_OH) and then diluted further in PBS as suggested by the manufacturer. Cells were treated with Aβ (1–40) or Aβ (1–40) HiLyte at the concentration of 100 nM in complete medium.

PAI039 (Tiplaxtinin, Catalog # PZ0295) was purchased from Millipore Sigma, Burlington, MA, USA). PAI039 is a potent and selective Serpine-1 inhibitor [34] and demonstrated efficacy in vivo in multiple models of acute arterial thrombosis. A 20 mM stock solution was prepared in DMSO. In a typical experiment, NPCs were cotreated with isolated EVs and/or 2 μM PAI039 for 24 h. Literature indicates that 1 μM PAI-039 can effectively inhibit Serpine-1 activity in vitro [35]. PAI039 exerts its activity by binding close to the vitronectin binding site [36].

### Treatment of brain endothelial cells and EV isolation

Confluent HBMEC were exposed to HIV-1, Aβ (1–40) and/or Aβ (1–40) HiLyte for 48 h. EVs were isolated from the media using ExoQuick-TC exosome precipitation solution (System Biosciences, Mountain View, CA) according to the manufacturer’s specifications. Briefly, 10 ml culture medium from 1.7 × 10^7^ cells at confluency cultured in a P100 dish was centrifuged at 3000 g for 15 min to remove cells and debris. Then, the samples were mixed thoroughly with 2 ml of Exo-Quick exosome precipitation solution and incubated overnight at 4°C. The next day, the samples were centrifuged at 1500 g for 30 min; the supernatants were removed and centrifuged again at 1500 g for 5 min. The EV pellets were resuspended in 400 μl PBS and used for further studies. The aliquots of 20 μl of EV suspension for every 100 μl of cell culture media was used for NPC treatment.

### Transfection of brain endothelial cells

HBMEC were transfected with the CD63 RFP and Serpine-1 GFP constructs (Vectorbuilder, Chicago, IL, USA) using Purefection Transfection Reagent (System Biosciences) following the manufacturer’s protocol. Twenty-four hours post transfection, cells were exposed to HIV-1 or/and Aβ (1–40) HiLyte for 48 h followed by EV isolation from the media. We have observed that even minor changes of HIV-1 stock preparation can lead to differences in EV release from HBMEC exposed to HIV-1. If HEK293T cells were cultured in Exo-FBS, their morphology changed, and the resulting viral stock had vastly different effects on EV release and EV isolation. Therefore, HEK293T cells were cultured in media containing regular FBS consistent with our previous reports [20, 22]. As a control for HIV-1 exposure, isolates from mock-transfected HEK293T cells were used.

### Nanoparticle tracking analysis (NTA)

EVs were analyzed by NanoSight model NS300 (Malvern Instruments Company, NanoSight, Malvern, United Kingdom) as described earlier [20]. Briefly, EV pellet samples obtained in the process of EV isolation were resuspended in 4% paraformaldehyde-PBS and further diluted 50-fold in PBS for analysis. During analysis, five 15 s videos were recorded for each sample. The obtained data were analyzed using Nanosight NTA Analytical Software (Malvern Instruments Company) with the detection threshold optimized for each sample and screen gain at 10 to track the maximal number of particles with minimal background.

### EV-Aβ HiLyte transfer assay

EV-derived Aβ HiLyte fluorescence was quantified in the recipient NPCs using a plate reader. Briefly, NPCs were plated in 96-well black plates (9,000 cells/well) and differentiated for 3 days with the last 24 h in the presence of the isolated EVs. To block Serpine-1 activity, selected NPC cultures were cotreated with 2 μM PAI039 and brain endothelial EVs for 24 h. After treatment, cells were washed with PBS, fixed with ethanol for 30 min at 4°C, washed again with PBS and Aβ HiLyte fluorescence was measured with a plate reader (Molecular Devices, SpectraMax iD3) as suggested by the manufacturer (Anaspec, Ex/Em 503/528 nm). Cell nuclei were stained with DRAQ5 (Cell Signaling, Catalog #4084L, dilution 1:1000) for 5 min, washed with PBS and DRAQ5 fluorescence was measured with the same plate reader (Ex/Em 647/681). Aβ HiLyte fluorescence was then normalized to nuclear DRAQ5 fluorescence.

### Enzyme Linked Immunosorbent Assay (ELISA) and Serpine-1 activity assay

ELISA was used to determine levels of human Serpine-1 (PAI-1) (Catalog # DSE100, R&D Systems, Minneapolis, MN, USA) in the parent cells, isolated brain endothelial EVs, NPC cell culture media, and NPCs.

Serpine-1 (PAI-1) activity was determined using the Plasminogen Activator Inhibitor Type 1 Human Chromogenic Activity Assay Kit (Abcam, #ab108894) according to the manufacturer’s instructions. Briefly, a fixed amount of tissue-type plasminogen activator (tPA) was added in excess to undiluted sample, which allowed Serpine-1 and tPA to form an inactive complex. The assay measures plasminogen activation by residual tPA in coupled assays that contain tPA, plasminogen, and a plasmin-specific synthetic substrate. The amount of plasmin produced was quantitated using a highly specific plasmin substrate releasing a yellow para-nitroaniline chromophore. The absorbance of the chromophore at 405 nm was inversely proportional to the Serpine-1 enzymatic activity. One arbitrary unit (AU) of inhibition was defined as the amount of Serpine-1 that can inhibit one IU of tPA/ml under the testing conditions. EV-Serpine-1 activity was also measured in the presence of Tx-100 1%, which was used to lyse EVs.

### Tissue plasminogen activator (tPA) assay

tPA activity was measured with the Tissue type Plasminogen Activator Activity Assay Kit (Abcam, #ab108905) following the manufacturer’s instructions. Briefly, the tPA assay protocol measures the ability of tPA to activate the plasminogen to plasmin in coupled or indirect assays that contain tPA, plasminogen, and a plasmin-specific synthetic substrate. The amount of plasmin produced was quantified using a highly specific plasmin substrate releasing a yellow para-nitroaniline chromophore. The change in absorbance of the chromophore in the reaction solution at 405 nm was directly proportional to the tPA enzymatic activity.

### Confocal microscopy

HBMEC cultured on collagen I coated chambered glass slides (Becton Dickinson Biosciences, San Jose, CA) were transfected with the CD63 RFP and Serpine-1 GFP constructs as described above. The parent cells expressing CD63 RFP and Serpine-1 GFP were imaged live after 24 h using an inverted confocal laser-scanning microscope (Olympus Fluoview 3000). Twenty-four hours post transfection, cells were exposed to HIV-1 or/and Aβ (1–40) HiLyte 647 for 48 h. At the end of treatment, the culture media was removed for fluorescent EV isolation; cells were washed with PBS and fixed with ethanol for 30 min at 4°C. CD63 RFP, Serpine-1 GFP and Aβ (1–40) HiLyte 647 fluorescence was imaged with an inverted confocal laser-scanning microscope (Olympus Fluoview 3000, objective lens UPLXAPO100XO 100X oil, numerical aperture 1.45) and analyzed using CellSens software.

After isolation from the media, fluorescent EVs were pipetted onto cleaned glass slides, heat fixed for 10 min at 95°C, and then fixed with ethanol for 30 min at 4°C followed by PBS wash. Slides were mounted using ProLong Gold Antifade reagent with or without 4’,6-diamidino-2-phenylindole (DAPI, Invitrogen, Carlsbad, CA, USA) to visualize the nucleic material in EVs. Specimens were covered with coverslips and the fluorescent images were evaluated and captured under confocal microscopy. Red fluorescence originating from EV-CD63 RFP, blue fluorescence from DAPI, green fluorescence from EV-Serpine-1 GFP and far-red fluorescence from EV-Aβ HiLyte-Alexa Fluor 647 was acquired directly using confocal microscopy (Olympus, Fluoview 3000, 100x oil immersion lens, room temperature). Serpine-1 GFP positive, CD63 RFP positive EVs were counted with the CellSens software and expressed as percentage of the total DAPI positive EVs. Serpine-1 GFP and CD63 RFP double positive EVs were counted and expressed as percentage of the total Serpine-1 GFP positive EV number.

NPCs were seeded on laminin coated 8-well chambered glass slides (15,000 cells/well) and incubated overnight at 37°C in maintenance culture medium (Millipore), containing 20 ng/ml FGF-2 and 20 ng/ml of rhEGF. The following day, the medium was changed to maintenance medium without growth factors to induce differentiation. Cells were allowed to differentiate for a total of 3 days, with the last 24 h in the presence of the isolated EVs containing Serpine-1 GFP, CD63 RFP +/− Aβ (1–40) HiLyte 647. After 1 h of EV exposure, CD63 RFP, Serpine-1 GFP and Aβ (1–40) HiLyte 647 fluorescence in the acceptor non-fluorescent NPCs was imaged live with an inverted confocal laser-scanning microscope. After 24 h of EV treatment, NPCs media was removed, cells were washed with PBS and fixed with ethanol for 30 min at 4°C. CD63 RFP, Serpine-1 GFP and Aβ (1–40) HiLyte 647 fluorescence in the acceptor non-fluorescent NPCs was imaged randomly as above.

HBMEC were exposed to HIV-1 or/and Aβ (1–40) HiLyte 488 for 48 h followed by EV isolation from the conditioned media. NPCs cultured on chambered slides and differentiated as above, were exposed to EV-Aβ HiLyte 488 for 24 h. Then, the media was removed, cells were washed with PBS and fixed with ethanol for 30 min at 4°C. Cells were washed again with PBS and transferred Aβ HiLyte 488 fluorescence was assessed by confocal microscopy.

On some confocal images we have increased brightness or contrast for better visibility of the fluorescence signal. These changes were consistent across all treatment groups to preserve the integrity of the data.

### Mitochondrial network analysis (MiNA)

Mitochondria were visualized with Mitotracker Deep Red (Invitrogen, Catalog #M22426). The total area of fluorescence intensity on the acquired images was normalized to the number of NPC nuclei. Confocal microscopy images were analyzed using FIJI software (v. 2.3.0, NIH). Images were pre-processed using Bio-Formats (v.6.9.0, https://github.com/ome/bioformats). Mitochondrial network parameters were analyzed using the Stuart Lab Mitochondrial Network Analysis (MiNA) (v. 3.0.1, https://github.com/StuartLab) plugin [37].

### Mitochondrial stress Seahorse assay

The Seahorse XFe24 Analyzer was used to calculate oxygen consumption rate (OCR; a measure of mitochondrial respiration) and extracellular acidification rate (ECAR; a measure of glycolysis) in NPCs using the Agilent Seahorse XF Cell Mito Stress Test Kit (Agilent Technologies, Santa Clara, CA, USA). Cells were seeded in a 24XF cell culture microplate at 30,000 cells/well differentiated and treated with brain endothelial EVs as described above. After EV treatment for 24 h, the cell culture media were replaced with 500 μl of the prepared Seahorse medium (containing Seahorse XF DMEM media, 2 mM L-glutamine, 10 mM glucose, and 1 mM pyruvate) and incubated at 37°C for 1 h. Measurements of mitochondrial respiration and glycolysis were carried out as previously described [38]. In brief, cells were treated with 1.5 μM concentrations of oligomycin (ATP synthase inhibitor of complex V), 1 μM carbonyl cyanide-p-trifluoromethoxyphenylhydrazone (FCCP, electron transport chain (ETC) uncoupler), and 0.5 μM rotenone with antimycin A (both ETC inhibitors of complex I and III, respectively) throughout the analysis. These treatments, which were added to the cells at specified time points, allowed for calculations of mitochondrial respiratory parameters, such as baseline OCR, ATP production, maximal respiration, proton leak, and non-mitochondrial oxygen consumption. After three basal measurements of OCR and ECAR were recorded, oligomycin was injected to inhibit ATP synthase and two more measurements were recorded to assess proton leak. Next, FCCP was injected to uncouple respiration and measure maximal respiration. Finally, antimycin A was injected to measure non-mitochondrial respiration. All OCR measurements were normalized to non-mitochondrial respiration and the final values normalized to NPC protein concentration in each well. Reserve capacity is the difference between maximal respiration and basal respiration, while ATP-linked OCR is the difference between basal and proton leak. The data were analyzed using the Wave Software (Agilent Technologies). All conditions were measured in 4–6 samples/group, and three repeats were performed for this experiment.

### Expression of synaptic proteins

EV-treated NPCs cultured on laminin coated chambered glass slides (ibidi USA, Madison, WI, USA) were fixed with ethanol for 30 min at 4°C. After washing with PBS and blocking with 3% bovine serum albumin in PBS for 30 min at room temperature, samples were incubated overnight at 4°C with the primary antibody: mouse anti-PSD95 monoclonal antibody (Abcam, Waltham, MA, USA, Catalog #192757, 1:1000) or rabbit anti-synaptophysin polyclonal antibody (Abcam, 1:1000). Then, the excess of primary antibody was removed, slides were washed with PBS, and incubated with Alexa Fluor 488/594-conjugated secondary antibodies (1:1000, Invitrogen) for 2 h at room temperature. Nuclei were stained with Hoechst 33342 (Invitrogen, Catalog #H3570). The immunofluorescent images were evaluated and captured under confocal microscopy. Red fluorescence originating from PSD95, blue fluorescence from Hoechst 33342, and green fluorescence from synaptophysin was acquired directly using confocal microscopy (Olympus, Fluoview 3000, 100x oil immersion lens, room temperature).

Quantitative analysis of synaptic protein expression was performed similarly to a previously published method, with modifications [39]. After immunofluorescence staining for PSD95 and synaptophysin, fields were selected randomly using the guidance of DAPI nuclear staining (three images/treatment group, total 9 images from three independent experiments), then confocal images were taken. On each confocal image (bright field), 20 identical rectangular areas were randomly superimposed on different segments of NPC projections. The selected areas were analyzed for green (synaptophysin) and red (PSD95) fluorescence intensity using the NIH Image J software (Bethesda, MD, USA). Mean Fluorescence Intensity (MFI) for each area was normalized by subtracting the background fluorescence intensity for that image. Synaptic protein densities were expressed relative to those in control NPCs. For total synaptophysin and PSD95 assessment, mean fluorescence intensity on the acquired images was normalized to the number of NPC nuclei.

### Statistical analysis

Data were analyzed using GraphPad Prism 9.0 (Graphpad Software, San Diego, CA). ANOVA was used to compare responses among treatments. Treatment means were compared using All Pairwise Multiple Comparison Procedures and p < 0.05 was considered significant.

## Results

### Serpine-1 is concentrated in EVs released from control and HIV-1 exposed brain endothelial cells

**Figure 1:**
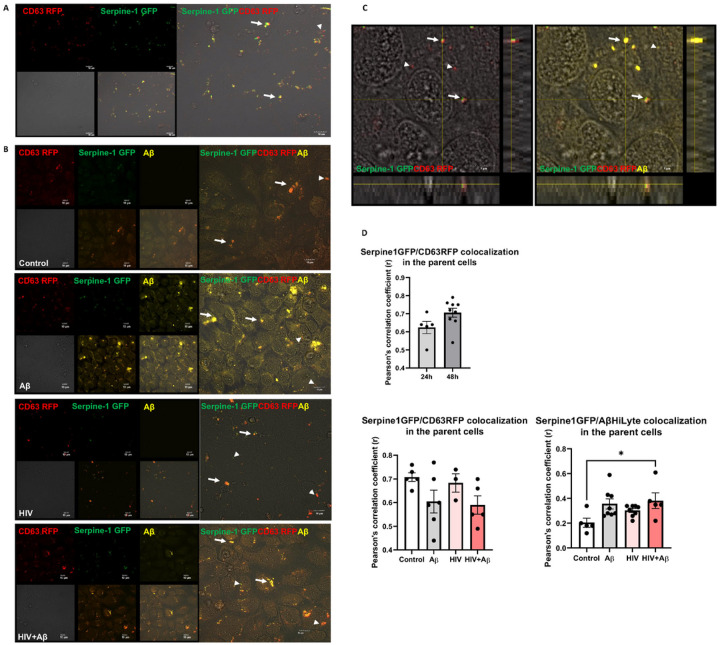
Serpine-1 is concentrated in vesicular structures in brain endothelial cells HBMEC were transfected with the Serpine-1 GFP and CD63 RFP plasmids (A) and 24 h after transfection, cells were exposed to HIV-1 (30 ng p24/ml) and/or 100 nM Aβ (1–40) HiLyte 647 for 48 h (B). (A) The images represent live imaging of Serpine-1 GFP and CD63 RFP (arrow heads) in the parent HBMEC 24 h after transfection. Scale bar: 10 μm. (B) Serpine-1 GFP, CD63 RFP, or Aβ HiLyte 647-positive fluorescence (arrow heads) in the fixed parent cells 48 h after vehicle (control), HIV-1, and/or Aβ exposure. Arrows indicate overlapping positive fluorescence of Serpine-1 GFP, CD63 RFP, and/or Aβ HiLyte 647. Scale bar: 10 μm. Representative images from four experiments. (C) Confocal z-stack imaging of Serpine-1 GFP, CD63 RFP, and Aβ HiLyte 647 colocalization(arrows) in the fixed parent cells 48 h after HIV-1 and Aβ exposure. Scale bar: 5 μm. (D) Quantification of colocalization of Serpine-1 GFP and CD63 RFP in the live parent cells after transfection (upper graph), unpaired t-test; Colocalization of Serpine-1 GFP and CD63 RFP (lower left graph) and Serpine-1 GFP and Aβ HiLyte 647 (lower right graph) in the fixed parent cells 48 h after HIV-1 and/or Aβ exposure. Values are mean ± SEM, n=3–9. One- and two-way ANOVA with Tukey’s multiple comparisons test. *Statistically significant at p<0.05.

Secretion of Serpine-1 in EVs was traced by cotransfection of HBMEC with the Serpine-1 GFP and CD63 RFP constructs. The tetraspanin CD63 is a membrane protein, which is predominantly localized to the vesicles, and; therefore, commonly used as a biomarker for EVs. As demonstrated by live fluorescence microscopy 24 h post transfection, transfected cells appeared to concentrate and secrete Serpine-1 GFP in CD63 RFP-positive EVs. Indeed, green fluorescence, corresponding to Serpine-1, noticeably overlapped with CD63 RFP-positive red fluorescent EVs budding off from the parent cells (arrow heads for single markers, arrows for overlapping fluorescence, Fig. 1A). We also measured the total number of EVs in the media originating from non-transfected HBMEC. EVs released from control HBMEC had a total EV concentration of 43.05 ± 6.65 × 10^8^ particles/ml, while EVs released from HIV-1 treated HBMEC had a total EV concentration of 83.40 ± 34.60 × 10^8^ particles/ml. This HIV-related increase in EVs release is consistent with our previous report [20].

Because both EVs and Serpine-1 were shown to play a role in Aβ pathology [20, 30, 31], we next evaluated the impact of HIV-1 on Serpine-1 and Aβ release via EVs. For these experiments, 24 h after cotransfection with Serpine-1 GFP and CD63 RFP, HBMEC were exposed to HIV-1 (30 ng p24/ml) and/or 100 nM Aβ (1–40) HiLyte 647 for 48 h. The treatment was terminated by removing the cell culture media for EV isolation, followed by washing with PBS and fixing the parent cells. As illustrated in Figs. 1B and 1C, Serpine-1 GFP green fluorescence and CD63 RFP red fluorescence partially overlapped with the fluorescent Aβ HiLyte 647 (yellow) taken up by the parent cells (arrow heads for single-, arrows for overlapping fluorescence). The images were quantified for Serpine-1, CD63, or Aβ HiLyte colocalization (Fig. 1D), indicating that Serpine-1 GFP and Aβ HiLyte 647 colocalization significantly increased in the HIV + Aβ group when compared to control (Fig. 1D, lower right graph).

**Figure 2:**
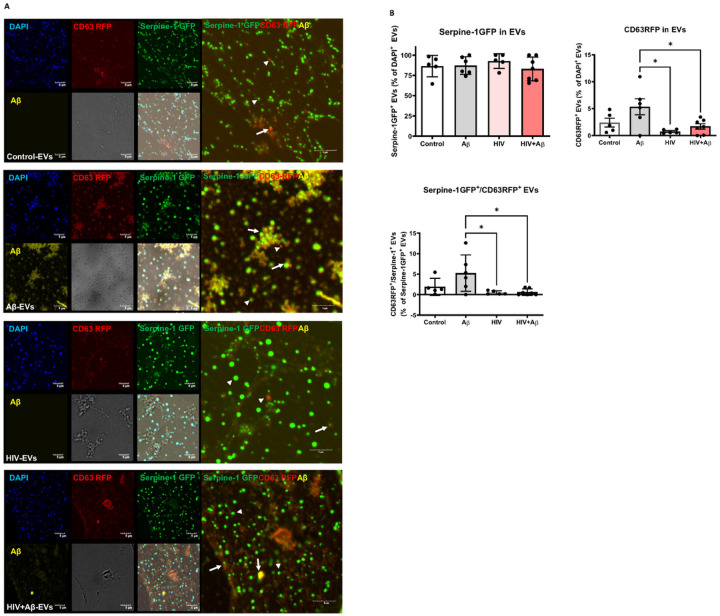
Serpine-1 is released in EVs from control, Aβ, and/or HIV-1 exposed brain endothelial cells (A) Visualization by confocal microscopy of Serpine-1 GFP (green), CD63 RFP (red), and Aβ (1–40) HiLyte (yellow) (arrow heads) in EVs isolated from media of transfected and treated HBMEC as in Figure 1. Examples of overlapping fluorescence of Serpine-1 GFP, CD63 RFP, and/or Aβ (1–40) HiLyte associated with EVs are indicated by arrows. DAPI stains the genetic material in EVs. Representative images from three experiments. Scale bar: 5 μm. (B) Quantification of Serpine-1 GFP-positive, CD63 RFP-positive, and Serpine-1 GFP/CD63 RFP-double positive EVs. Values are mean ± SEM, n=5–7. One- and two-way ANOVA with Šídák’s and Tukey’s multiple comparisons tests. *Statistically significant at p<0.05.

Next, Serpine-1 GFP, CD63 RFP and Aβ (1–40) HiLyte 647 fluorescence was visualized in EVs isolated from cell culture media. Serpine-1 GFP was detected in EVs of different sizes in control, Aβ and/or HIV-1 treated samples (Fig. 2A) indicating that the parent cells secrete Serpine-1 via EVs. Interestingly, overall fewer CD63-RFP positive EVs were present in all groups and Aβ HiLyte 647 occasionally colocalized with both Serpine-1 GFP and CD63-RFP in the secreted EVs. In the Aβ groups we frequently observed aggregates of Aβ HiLyte 647 with associated EVs (Fig. 2A, arrow heads for single markers, arrows for overlapping fluorescence). We also stained the EV genetic material with DAPI (blue fluorescence). Most of the EVs showed DAPI fluorescence indicating DNA/RNA cargo.

To further characterize the isolated EVs, we counted the number of Serpine-1 GFP-, CD63 RFP− and DAPI positive EVs from the confocal microscopy images. As quantified on the graphs from Fig. 2B, most of the DAPI containing EVs were also positive for Serpine-1 GFP. This percentage did not change significantly in any treatment group. In contrast, the percentage of CD63 RFP positive EVs was much lower and significantly decreased in the HIV-EV and the HIV + Aβ-EV groups as compared to the Aβ-EV group. Similarly, the number of Serpine-1 GFP and CD63 RFP double positive EVs changed in the same way (Fig. 2B).

### HIV-1 impacts Serpine-1 levels and activity in the released EVs

**Figure 3:**
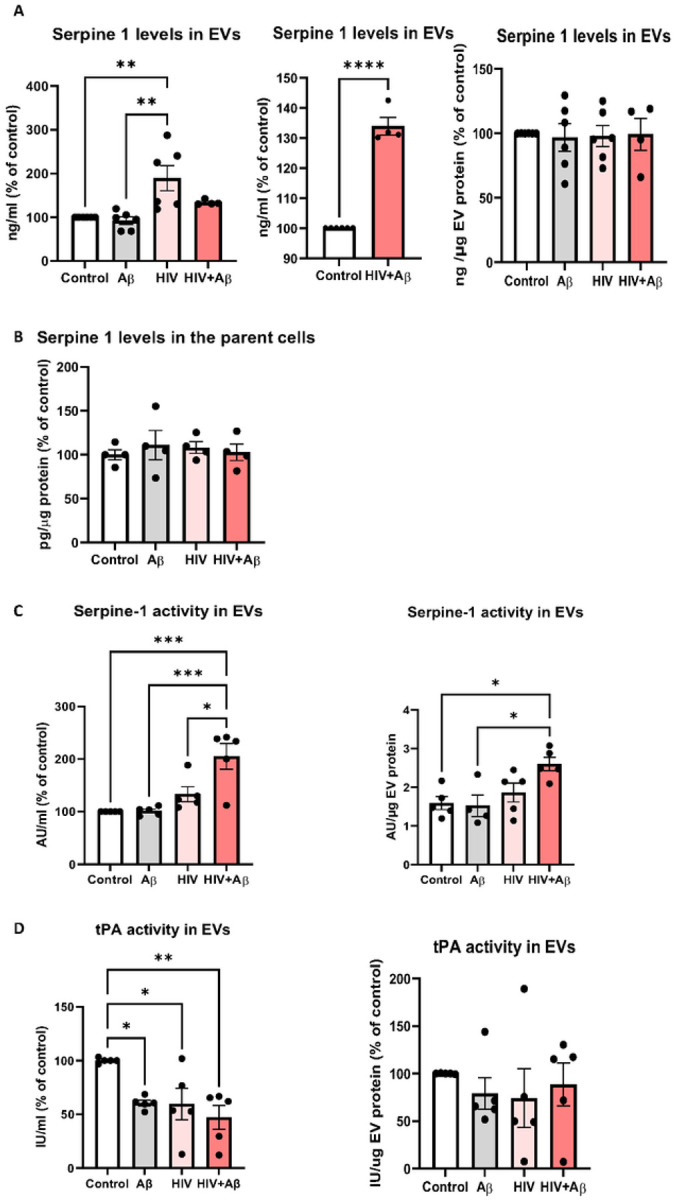
Serpine-1 levels and activity in the released EVs. Non-transfected HBMEC were exposed to HIV-1 (30 ng p24/ml) and/or 100 nM Aβ (1–40) for 48 h, followed by isolation of released EVs from the culture media. Serpine-1 levels in EVs and the parent cells were measured by ELISA. Serpine-1 activity and tPA activity were determined by specific activity assays. (A) Serpine-1 levels in isolated EVs normalized to cell culture media volume (left and middle graph) or to EV protein content (right graph). (B) Serpine-1 levels in the parent cells. Values are mean ± SEM, n=4–8. (C) Serpine-1 activity in the isolated EVs normalized to cell culture media volume (left graph) or to EV protein content (right graph). (D) tPA activity in the isolated EVsnormalized to cell culture media volume (left graph) or to EV protein content (right graph). Values are mean ± SEM, n=4–5. One- and two-way ANOVA with Tukey’s multiple comparisons test. *Statistically significant at p<0.05, **p<0.01, ***p<0.001, or ****p<0.0001.

Serpine-1 levels in the EV lysates were next assessed by ELISA in non-transfected HBMEC and normalized either to cell culture media volume or to EV protein content (Figs. 3A). EVs isolated from control, Aβ, and/or HIV-treated HBMEC cultures contained Serpine-1. Importantly, Serpine-1 levels were significantly higher in the HIV-1 group as compared to the control or Aβ groups when normalizing to cell culture volume (Fig. 3A, left graph). Serpine-1 levels in the HIV + Aβ group were also significantly increased when compared only to control (Fig. 3A, middle graph). Nevertheless, EV Serpine-1 concentration was similar in all treatment groups after normalization to EV protein levels (Fig. 3A, right graph), presumably because exposure to HIV-1 increases the overall EV number produced as reported before [20]. Interestingly, Serpine-1 levels in the parent cells were in the picogram range and thus much lower as compared to the nanogram range of Serpine-1 in EVs. Moreover, Serpine-1 levels in the parent cells did not change after HIV-1 and/or Aβ treatment (Fig. 3B).

Serpine-1 is an enzyme, therefore, its activity was also measured from the isolated EVs lysed with 1% Tx100 (see Methods). HIV + Aβ-EVs had the highest Serpine-1 activity, which was statistically significant when compared to the control and Aβ groups and remained significant even after normalizing the results to EV protein content (Fig. 3C).

Because Serpine-1 is known to inhibit tPA activity [24]; therefore, we also measured tPA activity in the isolated EVs. As illustrated on Fig. 3D, tPA activity trends were the opposite of those of Serpine-1 activity. Treatment with Aβ, HIV-1 and HIV + Aβ of the parent cells had a significant impact on tPA activity in EVs, which significantly decreased in these groups as compared to control, when normalizing to cell culture volume (Fig. 3D, left graph). A decreasing trend was also observed when normalizing the results to EV protein content (Fig. 3D, right graph) but the changes were not statistically significant.

### HBMEC-derived EVs transfer Serpine-1 cargo to recipient neural progenitor cells

HBMEC are part of a functional unit at the BBB consisted of pericytes, perivascular astrocytes, microglia, and neurons, called the neurovascular unit [40]. Therefore, we hypothesized that HBMEC-derived EVs can transfer Serpine-1 to neighboring cells of the neurovascular unit, including neural progenitor cells (NPCs). It was shown that ~ 47% of dividing progenitor and 46% of transit amplifying cells (precursors of neuroblasts) are located in close proximity to the brain endothelium [41, 42].

In order to assess Serpine-1 transfer to NPCs, HBMEC transiently transfected with Serpine-1 GFP and CD63-RFP were exposed to 100 nM Aβ HiLyte 647 and/or HIV-1 for 48 h, resulting in secretion of Serpine-1 GFP positive and CD63 RFP positive EVs, with some of them containing fluorescent Aβ cargo. EVs were isolated from the cell culture media and then employed to differentiating NPCs for 24 h. Green fluorescence signals (corresponding to EV-derived Serpine-1 GFP), red fluorescent signals (indicating EV-CD63 RFP) and yellow Aβ HiLyte 647 fluorescence (indicating EV-Aβ cargo) in the acceptor non-fluorescent NPCs was assessed by confocal microscopy.

**Figure 4:**
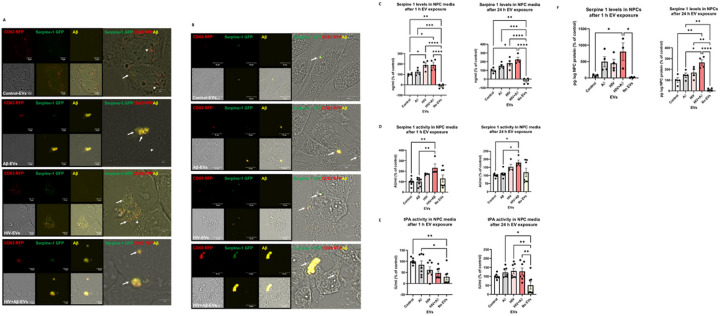
Transfer of Serpine-1 from donor HBMEC-derived EVs to recipient neural progenitor cells (NPCs). HBMEC were transfected and treated as in Figure 1, followed by isolation of EVs from the cell culture media and treatment of NPCs for up to 24 h. All images were performed by confocal microscopy. (A) Live imaging of Serpine-1 GFP (green), CD63 RFP (red) and Aβ (1–40) HiLyte (yellow) in non-fluorescent NPC cultures (arrow heads) after 1 h exposure to EVs from Control HBMEC, HIV-1 and/or Aβ-treated HBMEC. Scale bar: 5 μm. (B) Visualization of Serpine-1 GFP (green), CD63 RFP (red) and Aβ (1–40) HiLyte (yellow) in fixed non-fluorescent NPC cultures (arrow heads) after 24 h of EVs exposure. Examples of overlapping fluorescence of Serpine-1 GFP, CD63 RFP and Aβ (1–40) HiLyte in (A) and (B) are indicated by arrows. Scale bar: 10 μm. Representative images from three experiments.(C-F) Non-transfected HBMEC were treated as in Figure 3. EVs were isolated from the culture media and employed for NPC treatment for 1 (left panels) or 24 h (right panels). Serpine-1 levels (C), Serpine-1 activity (D), and tPA activity (E) were determined in NPC mediaas in Figure 3. (F) Serpine-1 levels in the recipient NPCs 1 h (left graph) and 24 h (right graph) after EVs exposure Values are mean ± SEM, n=4–7. One- and two-way ANOVA with Tukey’s multiple comparisons test. *Statistically significant at p<0.05, **p<0.01, ***p<0.001, and ****p<0.0001.

Representative images of NPC cultures exposed for 1 h to EVs derived from control, HIV plus/or Aβ-treated HBMEC are illustrated in Fig. 4A, with a variety of vesicular and non-vesicular structures. Some EVs show red fluorescence due to the presence of the EV marker CD63 RFP. EVs also exhibit yellow fluorescence indicating Aβ transfer via EVs derived from Aβ-treated HBMEC. Some of the EVs with fluorescent Aβ cargo show an overlapping red or green fluorescence indicating colocalization with Serpine-1 and/or CD63 (Fig. 4A, arrow heads for individual-, arrows for overlapping fluorescence). After 24 h EV exposure, NPC media was removed, and the acceptor NPCs were fixed and imaged again. Figure 4B visualizes Serpine-1 GFP, CD63 RFP and Aβ HiLyte transfer to NPCs by EVs derived from HIV and/or Aβ HiLyte-exposed HBMEC. Some of the transferred fluorescent Aβ appeared to be concentrated in large yellow aggregates (arrows), particularly in the HIV + Aβ group (Fig. 4B).

We also quantified HBMEC-derived EV-Serpine-1 cargo transfer to NPCs. For these experiments, non-transfected HBMEC were exposed to 100 nM non-fluorescent Aβ and/or HIV-1 for 48 h. EVs were isolated from the cell culture media and used for the subsequent NPC exposure for 24 h, followed by PBS wash. First, Serpine-1 levels in the NPC culture media were assessed by ELISA 1 h and 24 h after EV exposure (Fig. 4C). As illustrated, 1 h after EV exposure, Serpine-1 levels were significantly higher in the HIV-EV and HIV + Aβ-EV treated groups as compared to the Control-EV group. In addition, Serpine-1 levels in all EV-treated groups were significantly higher when compared to the No EV group, indicating that Serpine-1 in the NPC culture media, indeed, originated from the HBMEC-derived EVs and was not secreted by the recipient NPCs. This trend was maintained at 24 h after EVs exposure as well, indicating that most of the Serpine-1 was still present in the culture media and originated from the employed EVs (Fig. 4C, right graph).

Next, NPC culture media samples were used to assess Serpine-1 activity 1 h and 24 h after EVs treatment (Fig. 4D). Although Serpine-1 activity measurements were low, they showed a statistically significant increase in the HIV + Aβ-EV group when compared to the control-EV group, corresponding to Serpine-1 levels measured by ELISA. An increasing trend in the HIV-EV group was also observed although values in the No EV group were scattered (Fig. 4D).

In the following experiments, we explored whether Serpine-1 in the NPC media can inhibit tPA activity. As illustrated on Fig. 4E (left graph), tPA activity after 1 h was the highest in the control-EV group and showed a decreasing trend in the other groups. tPA activity in the Control-EV and Aβ-EV groups was significantly higher as compared to the No EV group (Fig. 4E). Overall, tPA activity change trends were the opposite of the Serpine-1 activity trends, confirming that the transferred EV-Serpine-1 can inhibit the transferred EV-tPA activity. Interestingly, 24 h after EV exposure, tPA activity levels were similar in all EV-treated groups and most of them were significantly higher as compared to the No EV group (Fig. 4E, right graph), verifying that tPA activity in NPCs originated mainly from the employed EV exposure and that NPCs have no or minimal endogenous tPA activity.

Serpine-1 levels and activity were also measured in the recipient NPCs. As shown on Fig. 4F, Serpine-1 levels were much lower (picogram range) in the recipient NPCs as compared to the media (nanogram range). These levels were significantly higher in the HIV + Aβ-EV treated group as compared to the Control-EV group and to the No EV group, again indicating that Serpine-1 in the NPCs originated from the HBMEC-derived EVs. This trend was similar at 1 and 24 h after EVs exposure (Fig. 4F). Finally, we measured Serpine-1 activity and tPA activity in the recipient NPCs 1 h and 24 h after EVs exposure; however, the levels were mostly very low or undetectable (data not shown). These results are consistent with Serpine-1 and tPA being secreted enzymes; therefore, they may primarily exert their effects on the recipient NPCs from the extracellular side.

### Serpine-1 is involved in EV-mediated transfer of Aβ cargo to recipient NPCs

**Figure 5:**
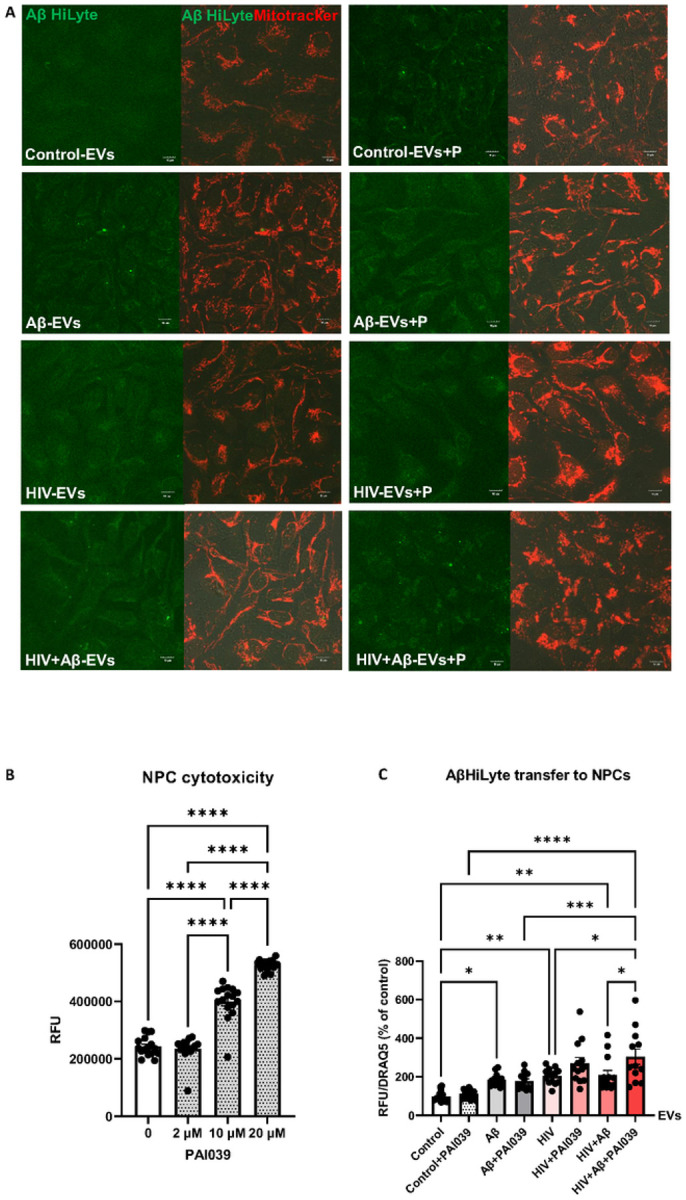
Involvement of Serpine-1 in the transfer of Aβ cargo from HBMEC-derived EVs to recipient NPCs. HBMEC were exposed to HIV (30 ng/ml) and/or 100 nM Aβ (1–40) HiLyte 488 for 48 h, followed by isolation of EVs from the cell culture media and treatment of NPCs for 24 h in the presence or absence of PAI039. (A) Confocal images of recipient NPCs with Aβ HiLyte fluorescence (green) and Mitotracker (red). (B) Dose-dependent PAI039 cytotoxicity in NPCs. (C) Quantification of Aβ HiLyte fluorescence in recipient NPCs. NPCs grown on 96-well plates were exposed to HBMEC-derived fluorescent EVs for 24 h. Controls were exposed to non-fluorescent EVs from HBMEC. Selected NPCs were cotreated with the Serpine-1 inhibitor PAI039 (2 μM) and EVs for 24 h. After washing with PBS, Aβ HiLyte fluorescence was measured (Abs/Em 503/528 nm) in a plate reader. The values were normalized to nuclear DRAQ5 fluorescence. Values are mean ± SEM, n=12–14. One-, two- and three-way ANOVA with Tukey’s multiple comparisons test. *Statistically significant at p<0.05, **p<0.01, ***p<0.001, ****p<0.0001.

After establishing that HBMEC-derived EVs can transfer both Serpine-1 and Aβ, we explored if Serpine-1 can be involved in Aβ transfer and/or uptake by NPCs. Non-transfected HBMEC were exposed to 100 nM Aβ HiLyte 488 and/or HIV-1 for 48 h, followed by isolation of EVs, which were then employed for NPC exposure for 24 h. Representative images of NPCs exposed to fluorescent EVs for 24 h visualize the transferred Aβ HiLyte (green fluorescence) and the NPC mitochondria traced with Mitotracker (red fluorescence) (Fig. 5A). To assess the involvement of Serpine-1 in this process, NPCs were cotreated with the Serpine-1 inhibitor PAI039 for 24 h. PAI039 was used at low concentration of 2 μM, which did not affect NPC viability (Fig. 5B).

EV-derived Aβ HiLyte fluorescence was next quantified in the recipient NPCs using a plate reader and normalized to nuclear DRAQ5 fluorescence (Fig. 5C). A significant fluorescence increase was observed upon treatment with EVs derived from Aβ HiLyte-treated HBMEC and HIV-1 plus Aβ HiLyte-treated HBMEC as compared to treatment with EVs from control HBMEC. PAI039 affected the EV-Aβ HiLyte transfer only in NPCs exposed to EVs derived from HIV-1 plus Aβ HiLyte HBMEC. Specifically, cotreatment with PAI039 significantly increased Aβ transfer in this group as compared to the HIV + Aβ-EV group without inhibitor (Fig. 5C). These results indicated that transfer of EV-derived Aβ cargo could be modulated by Serpine-1 only in specific conditions, such as HIV-1 exposure.

When we incubated only the isolated EVs with the inhibitor, we have observed that PAI039 paradoxically increased EV-Serpine-1 activity in the HIV groups and caused an increasing trend in the non-HIV groups when compared to the respective groups without PAI039 (**Supplementary Fig. 1**). Although observed in a different experimental set-up, these unexpected effects may have contributed to the impact of PAI039 on EV-Aβ transfer to NPCs in the context of HIV-1. On the other hand, PAI039 did not change tPA activity in the isolated EVs (**Supplementary Fig. 1**).

### Serpine-1 impacts mitochondrial networks and functions in NPCs exposed to HBMEC-derived EVs

In the next series of experiments, we investigated the implications of Serpine-1 transfer to NPCs via EVs by evaluating the mitochondrial networks and functions. In support of this line of investigation, there is evidence that proper mitochondrial functions of NPCs are essential for correct neurogenesis [43].

**Figure 6:**
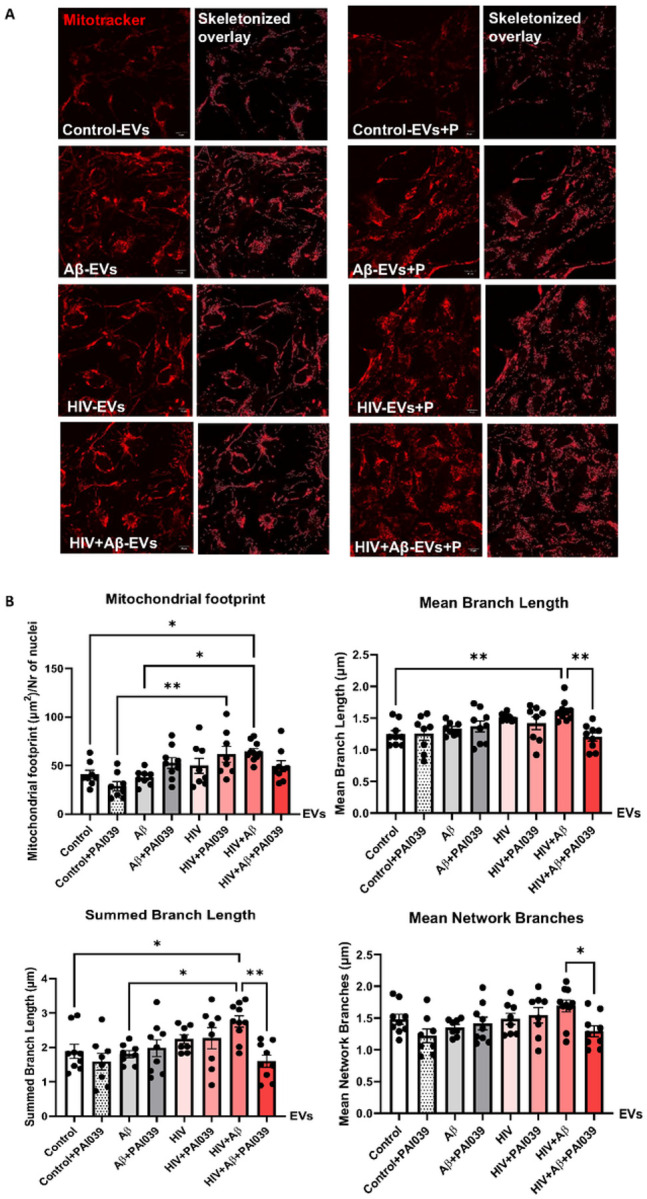
Mitochondrial network analysis (MiNA) of mitochondrial morphology in EV-exposed NPCs. Non-transfected HBMEC were treated as in Figure 3. EVs were isolated from the culture media and employed for NPC treatment for 24 h. Selected NPCs were cotreated with PAI039 (2 μM) and EVs for 24 h. (A) Confocal images of NPCs stained with Mitotracker Deep Red (red) for tracking the mitochondria. MiNA plugin on ImageJ was used to skeletonize the mitochondria. Scale bar: 10 μm. (B) Quantification of the mitochondrial footprint, mean branch length, total branch length and mean network branches. Values are mean ± SEM, n=8–10. One-, two- and three-way ANOVA with Tukey’s multiple comparisons test. *Statistically significant p<0.05, **p<0.01.

To assess mitochondrial morphology changes, we employed Mitotracker Deep Red to stain mitochondrial networks in the NPCs treated with EVs isolated from control, Aβ, and/or HIV-exposed HBMEC (Fig. 6A). The experiments also involved treatment with PAI039. Morphological analysis was performed using mitochondrial network analysis (MiNA), an Image J plug-in, as previously published [37] to quantify the networks pre-processed and skeletonized by the software (skeletonized images on Fig. 6A and graphs on Fig. 6B). Mitochondrial footprint (the total area of the image with the Mitotracker fluorescence signal, normalized to the number of nuclei), was significantly increased in the HIV + Aβ-EV group when compared to the Aβ-EV and control-EV groups. In addition, mitochondrial footprint was significantly higher in the HIV-EV + PAI039 group as compared to the control-EV + PAI039 group, indicating HIV-1-mediated impact (Fig. 6B, upper left graph). The mean branch length was also significantly increased in the HIV + Aβ-EV group when compared to the control-EV group, and this effect was significantly blocked by the PAI039 exposure (Fig. 6B, upper right graph). The presence of long branches may indicate hyperfusion, leading to an elongated branched network. The summed or total branch length showed a similar change with a significant increase in the HIV + Aβ-EV group when compared to the control-EV or Aβ-EV only groups and PAI039 abolished this effect (Fig. 6B, lower left graph). Consistent with these results, mean network branches were also significantly decreased in the HIV + Aβ-EV + PAI039 group when compared to the HIV + Aβ-EV group (Fig. 6B, lower right graph). Taken together, these results indicate that in the HIV + Aβ-EV group the mitochondrial branches are longer; however, PAI039 can reverse these effects.

**Figure 7:**
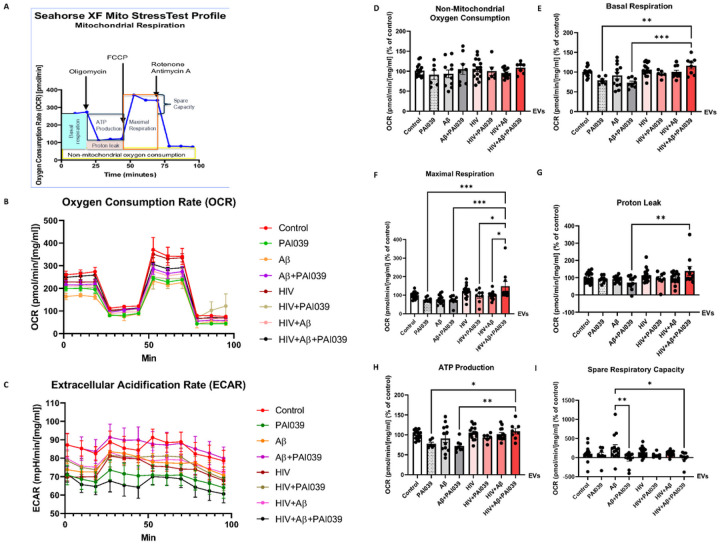
Mitochondrial functions in recipient NPCs after exposure to HBMEC-derived EVs. Non-transfected HBMEC were treated as in Figure 3. EVs were isolated from the culture media and employed for NPC treatment for 24 h in the presence or absence of the Serpine-1 inhibitor PAI039 (2 μM). (A) Seahorse Mitochondrial Stress Test diagram. (B) Oxygen consumption rates (OCR) and (C) Extracellular acidification rates (ECAR) in the treatment groups. (D) Non-mitochondrial oxygen consumption, (E) Basal respiration, (F) Maximal respiration, (G) Proton leak, (H) ATP production, (I) Spare respiratory capacity in the treatment groups are represented as % of the control. Representative graphs from three experiments. Values are mean ± SEM, n=6–18. One-, two- and three-way ANOVA with Šídák’s and Tukey’s multiple comparisons tests. *Statistically significant at p<0.05, **p<0.01, ***p<0.001.

Next, we assessed whether mitochondrial network changes were accompanied by functional changes as determined using the Seahorse Mito Stress assay. For these experiments, HBMEC were treated with Aβ and/or HIV, followed by isolation of EVs as in Fig. 3. Then, human NPCs seeded on 24-well Seahorse plates (30,000/well) were differentiated for 3 days with exposure to these isolated EVs for the last 24 h. Selected NPC cultures were also cotreated with PAI039 (2 μM) and EVs for 24 h. Mitochondrial oxidative phosphorylation was measured by the oxygen consumption rate (OCR) and glycolysis by analyzing the extracellular acidification rate (ECAR) in real-time in live NPCs (Fig. 7). Data from the Seahorse were normalized to NPC protein concentration per well.

Figure 7A depicts the Seahorse XF Mito Stress Test Profile, while Figs. 7B and C represent OCR and ECAR graphs, respectively. Non mitochondrial oxygen consumption did not change in NPCs after exposure to any EV groups (Fig. 7D). Interestingly, basal respiration was significantly higher in the NPC cultures exposed to EVs derived from HIV plus Aβ-treated HBMEC and cotreated with PAI039 as compared to cultures treated with PAI039 and Aβ-EVs or with PAI039 alone (Fig. 7E). Similar changes were observed for maximal respiration, with the highest values being recorded for the NPC cultures exposed to EVs derived from HIV plus Aβ-treated HBMEC and cotreated with PAI039 (Fig. 7F). Strikingly, maximal respiration in this group was significantly higher as compared to cultures treated with HIV + Aβ-EVs, HIV-EVs + PAI039, Aβ-EVs + PAI039, or control-EVs + PAI039 (Fig. 7F). Proton leak was significantly increased in the HIV + Aβ-EV + PAI039 group as compared to the Aβ-EV + PAI039 group (Fig. 7G). PAI039 did not change significantly the ATP production in the NPCs as compared to the corresponding treatment groups without PAI039. However, ATP production was significantly higher in the HIV + Aβ-EV + PAI039 group as compared to the Aβ-EV + PAI039 or control-EV + PAI039 groups (Fig. 7H). On the other hand, PAI039 significantly decreased spare respiratory capacity in the Aβ-EVs + PAI039 treated NPCs as compared to the Aβ-EVs only group (Fig. 7I). Although shown under different experimental conditions, the unexpected EV-Serpine-1 activity increase evoked by PAI039 (**Supplementary Fig. 1**) may have contributed, at least partly, to these effects.

Overall, the obtained results indicate a substantial impact of Serpine-1 inhibition on bioenergetics of mitochondria. While significant increases in basal and maximal respiration, elevated proton leak and ATP production were all observed in the HIV + Aβ + PAI039-EV group, a decrease in spare respiratory capacity in this group suggests that Serpine-1 inhibition may impair the ability of cells to respond to stress or metabolic demand.

### Serpine-1 affects synaptic protein expression in the developing NPCs

Literature reports on potential neurotoxicity of Serpine-1 are conflicting. There are indicators that Serpine-1 can block the damage of neuronal networks in vitro by increasing postsynaptic density protein 95 (PSD95) and synaptophysin [44, 45]. In addition, Serpine-1 was demonstrated to be neuroprotective against NMDA-induced neuronal death [46]. In contrast, Serpine-1 was reported to inhibit tPA-mediated neurite outgrowth in NPCs [47].

**Figure 8:**
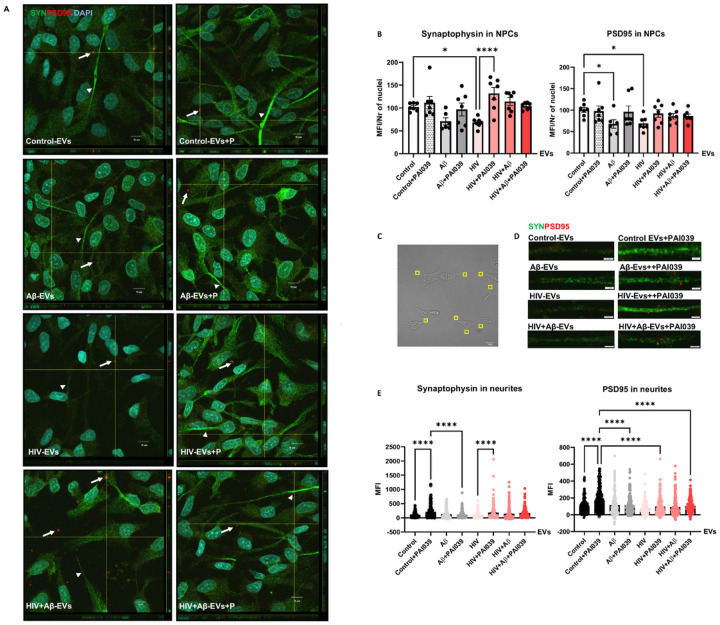
Impact of HBMEC-derived EVs on synaptic protein expression in NPCs. Non-transfected HBMEC were treated with HIV and/or Aβ and EVs were isolated as in Figure 3. Then, human NPCs were exposed to HBMEC-derived EVs for 24 h, with selected cultures additionally treated with 2 μM PAI039 (P) as in Figure 7. (A) synaptophysin (green; arrow heads) and PSD95 (red; arrows) immunoreactivity as imaged by confocal microscopy. DAPI staining (blue) visualizes the NPC nuclei. The combined z-stack images with maximum intensity projection are representative from three experiments. Scale bar: 10 μm. Individual panels are shown in Supplementary Figure 2. (B) Total intensity of synaptophysin (left graph) and PSD95 (right graph) immunoreactivity as quantified from the confocal images. Values are mean ± SEM, n=7. One-, two- and three-way ANOVA with Šídák’s multiple comparisons tests. (C) Example of a brightfield confocal image with random identical rectangular areas on NPC projections. (D) Synaptophysin (green) and PSD95 (red)immunoreactivity on representative confocal images of NPC projections (1 control-EVs, 2 control-EVs+PAI039, 3 Aβ-EVs, 4 Aβ-EVs+PAI039, 5 HIV-EVs, 6 HIV-EVs+PAI039, 7 HIV+Aβ-EVs, 8 HIV+Aβ-EVs+PAI039). Scale bar: 2 μm. (E) Intensity of synaptophysin and PSD95 immunoreactivity as quantified from random projection areas. Values are mean ± SEM, n=160–180. One-, two- and three-way ANOVA with Šídák’s and Tukey’s multiple comparisons tests. *Statistically significant at p<0.05, ****p<0.0001.

In order to get a better understanding of the involvement of Serpine-1 in synaptic protein expression in differentiating NPCs, we evaluated PSD95 and synaptophysin expression pattern in NPCs exposed to HBMEC-derived EVs in the presence or absence of PAI039. As illustrated on Fig. 8A and in **Supplementary Fig. 2**, synaptophysin exhibited a fine punctate immunoreactivity pattern (green fluorescence) in all NPC groups with strikingly stronger fluorescence in segments of the developing NPC projections (arrow heads). PSD95 showed a similar punctate immunoreactivity pattern (red fluorescence). In addition to this fine punctate pattern, PSD95 fluorescence was frequently observed in vesicular structures both inside and outside of NPCs in all groups (arrows) as revealed by z-stacking confocal microscopy (Fig. 8A). This was rarely the case for synaptophysin. We may speculate that PSD95 could possibly be also released via EVs, as was shown before [48], from the developing NPCs to be delivered to the developing synapses.

Quantification of total synaptophysin and PSD95 immunoreactivity from confocal z-stack images showed that both synaptic protein levels significantly decreased in NPCs exposed to HIV-EVs as compared to the Control-EVs treated group. This effect was reversed by PAI039 only for synaptophysin, indicating that PAI039 differentially affected synaptic protein levels overall (Fig. 8B). In addition, total PSD95 level was significantly decreased in NPCs after Aβ-EV exposure and PAI039 did not block this effect (Fig. 8B, right graph).

Next, we quantified synaptic protein fluorescence intensity in the NPC projections (Figs. 8C–E). Because these were developing NPCs and not mature neurons, their projections were short (Fig. 8C) and their synaptic protein immunofluorescence varied greatly from no signal to very high intensity signal even within the same neurite (Figs. 8A and 8D). Synaptophysin (green fluorescence) and PSD95 (red fluorescence) showed a similar punctate immunoreactivity pattern in the NPC projections, visible at high magnification (Fig. 8D). Interestingly, there appeared to be little overlap between synaptophysin and PSD95 signals in neurites on these high magnification images. Using an unbiased approach, we highlighted identical rectangular areas randomly on NPC projections visible on brightfield images as depicted on Fig. 8C. Then, we measured synaptophysin (green) and PSD95 (red) fluorescence intensity from the same areas. Quantification of these results are shown on Fig. 8E. Synaptophysin levels significantly increased in the Control-EV + PAI039 group as compared to the Control-EV group and in the HIV-EV + PAI039 group when compared to the HIV-EV only group. The Control-EV + PAI039 group was also significantly higher than the Aβ-EV + PAI039 group, suggesting that Aβ-EV treatment may have blocked the PAI039 effect (Fig. 8E, left graph). Changes in PSD95 fluorescence were partly similar, with PSD95 levels being significantly increased in the Control-EV + PAI039 group as compared to the Control-EV group. Interestingly, the Control-EV + PAI039 group was also significantly higher than the Aβ-EV + PAI039, HIV-EV + PAI039 and HIV + Aβ-EV + PAI039 groups, suggesting that Aβ and HIV may have blocked the PAI039 effect (Fig. 8E, right graph).

## Discussion

EVs are recognized as important contributors to Aβ pathology [49–55], including elevated Aβ deposition in HIV-1 infection [20, 22]. We have shown previously that EV-Aβ can be transferred to cells of the neurovascular unit, including neural progenitor cells (NPCs) [20]; however, the mechanisms of EV-mediated Aβ pathology remain elusive. In an effort to gain more insight into this process, we applied proteomics analysis to better characterize the EV protein cargo in the context of HIV-1, and Serpine-1 was identified as a main connecting “hub” on several EV protein-protein interaction maps [23]. This finding is important because Serpine-1 was previously described as a key player in Aβ pathology [30, 31] and was linked to HIV-1 infection as well [29, 56]. However, the role of EV-associated Serpine-1 in the Aβ pathology is not well understood. Since Serpine-1 was linked to Aβ pathology, HIV-1 comorbidities, and was found in EVs, we hypothesized that brain endothelial EV-Serpine-1 can be involved in HIV-1 and/or Aβ-induced NPC alterations. To the best of our knowledge, there are no reports in the literature on this subject.

The main physiological role of Serpine-1 is inhibition of tPA leading to inhibition of plasmin, which places Serpine-1 as the critical regulator of fibrinolysis pathways [24]. However, this process also affects Aβ levels because plasmin can degrade both APP and Aβ. Indeed, elevated levels of Serpine-1 favor a more procoagulant state by decreasing tPA activity, which, in turn, hinders plasminogen conversion to active plasmin leading to diminished Aβ degradation [28]. In support of this claim, Serpine-1 involvement in Aβ deposition in AD has been documented [30, 31]. For example, cerebral blood vessels were shown to be Serpine-1 positive in AD transgenic mice overexpressing Tau [57]. Knock-out of Serpine-1 gene or inhibition of Serpine-1 significantly reduced brain Aβ load in the APP/PS1 AD mouse model. Oral administration of TM5275, a small molecule inhibitor of Serpine-1, increased the activities of tPA, uPA and plasmin, leading to decreased Aβ levels in the hippocampus and cortex with improved learning and memory functions [31]. Moreover, Serpine-1 inhibition with PAI039 restored Aβ-induced decreased tPA activity and altered neurovascular coupling. These effects were associated with reduced perivascular amyloid deposition and improved cognition [58]. In related studies, tPA was proposed to protect against elevated Aβ levels by accelerating Aβ degradation and inhibition of Aβ-mediated neurodegeneration [28]. Overall, the literature data provide evidence that inhibition of Serpine-1 and restoration of tPA activity could be of substantial therapeutic value in AD.

Regarding a link to HIV infection, antiretroviral therapy (ART) was shown to affect plasma Serpine-1 levels, which appeared to be a marker for HIV-1 related comorbidities. For instance, HIV-1 infected patients on protease inhibitors had higher plasma Serpine-1 levels [56]. Moreover, high plasma levels of Serpine-1 were associated with high risk of myocardial infarction in HIV-1 infected people [29]. Elevated expression of Serpine-1 was proposed to be one of the mechanisms of HIV-1 protein Tat-induced inflammation in vascular cells [59], and synthetic Tat-derived peptides were demonstrated to increase production of Serpine-1 in human umbilical vascular endothelial cells [60]. In addition, monocytes from asymptomatic HIV positive viremic donors were characterized by significantly increased Serpine-1 protein levels as compared to HIV negative donors [61].

Earlier reports described the presence of Serpine-1 in microparticles in the blood [62], which might have been EVs as several recent publications reported its presence in EVs. For instance, proteomic analysis from trophoblast-derived EVs identified the presence of Serpine-1 [63]. EVs from nasal olfactory mucosa mesenchymal stem cells and EVs derived from ascites cell cultures also contained Serpine-1 [64, 65]. In line with these reports, we have confirmed that primary human brain endothelial cell-derived EVs (HBMEC-EVs) contained Serpine-1 [23] implicating the brain endothelium and the BBB as important contributors to the Serpine-1 pool. In experiments in which HBMEC were double-transfected with Serpine-1 GFP and CD63 RFP, we found that these cells concentrated and released Serpine-1 via EVs (Figs. 1 and 2). Interestingly, the isolated Serpine-1-positive EVs were rarely positive for CD63 RFP (Fig. 2B), suggesting that different sets of EVs with different cargoes were released by similar pathways from the parent cells, and Serpine-1 may be released in a specific set of EVs. Nevertheless, there is also a possibility that Serpine-1 GFP transfection efficiency was higher than that of CD63 RFP, although the amount of CD63 RFP plasmid DNA used was double of that of Serpine-1 GFP plasmid. We also observed that Serpine-1 GFP was associated with EVs of different sizes (Fig. 2A), underscoring the importance of evaluating total EVs in the HIV-related Aβ pathology as opposed to just a particular size-range EVs. Another interesting observation was that almost all Serpine-1 positive EVs were DAPI positive, which demonstrates their nucleic acid cargo (Fig. 2A). Strikingly, the number of CD63 RFP positive EVs was significantly lower in the HIV and HIV + Aβ groups as compared to the Aβ only group (Fig. 2B). Similar pattern was observed for the Serpine-1 GFP/CD63 RFP double positive EVs (Fig. 2B), suggesting that HIV-1 exposure of the parent cells alters the protein cargo of the released EVs.

In general, only low levels of Serpine-1 protein were described in the brain and our results are consistent with these observations as only low levels of Serpine-1 were detected in HBMEC. However, our important results indicate that Serpine-1 was concentrated in EVs, achieving ~ 30x higher levels when compared to the parent cells (Figs. 3A–3B). When comparing the treatment groups, EV-Serpine-1 levels were significantly higher in the HIV-1 and the HIV + Aβ groups than in controls when normalizing the data to cell culture volume (Fig. 3A). These results were consistent with the activity data (Fig. 3C). On the other hand, there were no differences between the groups when EV-Serpine-1 levels were normalized to EV protein content (Fig. 3A) because exposure to HIV-1 increases the overall EV number as published before [20]. Overall, these observations suggest that one of the main Serpine-1 pools in the brain may be found in endothelial-EVs, pointing to a critical role of the BBB in Serpine-1 related brain pathologies such as AD or stroke or even systemic pathologies as BBB-EVs may also be released into the peripheral circulation. Moreover, higher Serpine-1 levels in the HIV groups implicate EVs to creating a pro-coagulant environment in the vicinity of the BBB of HIV-1-infected brains. These observations are in line with the report that HIV-1 infection was associated with a more pro-coagulant state associated with high Serpine-1 levels in the plasma possibly increasing the risk of myocardial infarction [29]. These findings are also consistent with tPA activity being significantly reduced in the EV groups originating from HIV-1 and/or Aβ-treated HBMEC as compared to control EVs (Fig. 3D). Indeed, a decrease in tPA activity was observed to be consistently associated with elevated amyloid deposition in the brain [66].

One of the main biological functions of EVs is intercellular communication, which is executed by transferring cargo between different cells and cell types. We have shown before that endothelial-derived EVs can transfer Aβ to NPCs affecting their neurogenesis [22]. Knowing that Serpine-1 levels are concentrated in EVs, we next evaluated if EVs can serve as carriers to deliver Serpine-1 from endothelial cells to NPCs and what are the outcomes of this process. The rationale for these experiments is the fact that a large pool of NPCs is in the neurogenic niches of the perivascular space in direct proximity to the brain endothelium [42]. We confirmed that Serpine-1 could be transferred to NPCs via EVs (Fig. 4). Moreover, our results indicated that Serpine-1 is transferred together with Aβ cargo, potentially impacting the Aβ fate in the acceptor NPCs. Consistent with previously reported results, this process appeared to be enhanced in EVs derived from HIV-1-exposed HBMEC [20]. Nevertheless, Serpine-1 levels in acceptor NPCs were very low, suggesting that secreted EV-Serpine-1 acts on NPCs mostly extracellularly. This mode of action is consistent with the biological impact of Serpine-1 and tPA as both proteins are secreted and act in the extracellular environment.

To evaluate the interactions between Serpine-1 and Aβ, we examined whether Serpine-1 inhibition can affect EV-Aβ transfer to NPCs. Consistent with literature data [67], NPCs appeared to be sensitive to PAI039 toxicity, as 10 μM caused substantial toxicity (Fig. 5B) and 20 μM caused massive cell death; therefore, PAI039 was used at 2 μM. Inhibition of Serpine-1 had an unexpected effect on Aβ transfer to NPCs, enhancing this process in the HIV + Aβ-EV + PAI039 group as compared to the HIV + Aβ-EV group (Fig. 5C). The inhibitor did not affect Aβ transfer in other groups, suggesting a specific impact of Serpine-1 on the transfer of EV-derived Aβ cargo in the context of HIV-1. Although this was a unique complex system, where EVs interacted with NPCs, separate observations that PAI039 exposure with EVs alone increased Serpine-1 activity in the HIV groups (**Supplementary Fig. 1**) suggest that this phenomenon may have also affected EV-Aβ transfer.

The role of Serpine-1 in neuronal dysfunction is controversial with conflicting reports in the literature. It was observed that Serpine-1 might be neuroprotective against Aβ-induced neurotoxicity, preserving neuronal networks, and promoting synaptogenesis by increasing PSD95 and synaptophysin [44, 45]. Similarly, Serpine-1 was demonstrated to be neuroprotective against NMDA-induced neuronal death [46]. On the other hand, Serpine-1 can inhibit neuroprotective impact of tPA, which was shown to control neurite outgrowth in cortical neurons after stroke or in NPCs [47, 68]. This is an important observation, because both Aβ and HIV pathologies are linked to increased incidents of strokes and administration of tPA is an approved intervention to restore blood flow to brain regions affected by a stroke. By blocking these beneficial effects, Serpine-1 can exert undesirable neurotoxic impact. To address these problems, we evaluated the effects of EVs carrying Serpine-1 and/or Aβ on the NPC mitochondrial networks, bioenergetics, and synaptic integrity. The results clearly demonstrated altered mitochondrial morphology in NPCs exposed to HBMEC-derived EVs in the HIV + Aβ-EV treated group, with PAI039 reversing the majority of these alterations (Fig. 6B). The characteristic of these changes related to long mitochondria branches and an increase in total branch length may point to mitochondrial hyperfusion. In support of a role of EVs and HIV-1 in these mitochondrial alterations, EVs from latent HIV-infected T cells were shown to enhance mitochondrial superoxide production, reduce mitochondrial membrane potential, and induce mitochondrial hyperfusion in primary human brain microvascular endothelial cells [69]. In addition, the obtained results were consistent with the observations that Aβ may cause NPC damage with mitochondrial alterations, which, in turn, may affect their functions [43]. Regarding NPC mitochondrial bioenergetics, inhibition of Serpine-1 appeared to impair the cells’ ability to respond to stress or metabolic demand after Aβ-EV exposure as demonstrated by a decrease in spare respiratory capacity in this treatment group (Fig. 7F).

In order to assess the impact of Aβ and/or HIV-1 EVs on synaptic integrity, we evaluated the levels of synaptophysin and PSD95 in differentiating NPCs. Synaptophysin expression served as a marker of presynaptic plasticity and synaptogenesis [70]. Loss of synaptophysin was found in AD [71] and learning and memory deficits have been demonstrated in synaptophysin knockout mice [72]. PSD95 is a postsynaptic marker of synaptic integrity and its decrease can also lead to learning and memory impairment [73, 74]. In contrast, up-regulation of PSD-95 was shown to improve memory [75], underscoring the importance of PSD95 in these key brain functions. We detected that even in early developmental stage (namely, three days of differentiation), NPCs expressed a fine punctate immunoreactivity for both synaptophysin and PSD95 in the developing neurite segment (Fig. 8D). In addition, PSD95-positive, but not synaptophysin-positive, fluorescence was often demonstrated in vesicular structures both intra- and extracellularly on z-stacking confocal microscopy images (Fig. 8A), suggesting that it could be secreted via EVs from the developing NPCs. Exposure to EVs derived from HIV-exposed HBMEC decreased both synaptophysin and PSD95 in NPCs, which is consistent with neurotoxicity and aberrant neurogenesis in HIV infected brains [76, 77]. In addition, loss of synaptophysin was observed in HIV-1 infection in humanized mice [78] and exposure to HIV-1 proteins, like Tat and gp120, markedly decreased PSD95 in hippocampal neurons [79–81]. The levels of synaptophysin and PSD95 were also decreased in mice following exposure to HIV-1 Tat and methamphetamine [82]. Therefore, it was important that inhibition of Serpine-1 protected against HIV-EV-induced alterations in synaptophysin (Fig. 8B) and increased both synaptic proteins in the projections (Fig. 8E) of differentiating NPCs. These observations are consistent with the reports advocating for a beneficial effect of Serpine-1 inhibition on synaptic protein expression and neurite development, although EV-Serpine-1 activity increase evoked by PAI039 (**Supplementary Fig. 1**) may have also contributed to these effects. Overall, these data may be relevant in the context of HIV-1 associated neurocognitive impairments.

### Conclusions.

The results of the present study indicate that brain endothelial EVs contain active Serpine-1 cargo, which can be delivered to the recipient NPCs along with Aβ. These findings represent a novel concept that endothelial-derived EVs constitute a major Serpine-1 pool in the brain, which can create a pro-coagulant environment at the BBB and lead to mitochondrial and synaptic alterations in NPCs. These processes may further contribute to HIV-1 associated neurocognitive disorders (HAND), especially in older brains, which are characterized by elevated Aβ depositions.

## Data Availability

All source data supporting the findings of this manuscript are available from the corresponding authors upon request.

## Abbreviations

Aβ: amyloid beta
AD: Alzheimer’s disease
BBB: blood-brain barrier
bFGF: basic fibroblast growth factor
DAPI: 4’,6-diamidino-2-phenylindole
ECAR: extracellular acidification rate
EVs: extracellular vesicles
EGF: epidermal growth factor
ELISA: enzyme linked immunosorbent assay
HAND: HIV-associated neurocognitive disorders
GFP: green fluorescent protein
HBMEC: Human brain microvascular endothelial cells
HIV: human immunoDeficiency virus type 1
MiNA: mitochondrial network analysis
NPCs: neural progenitor cells
NTA: nanoparticle tracking analysis
OCR: oxygen consumption rate
PAI-1: plasminogen activator inhibitor 1
PBS: phosphate buffered saline
RFP: red fluorescent protein
Serpine-1: Plasminogen activator Inhibitor Type 1
PSD95: postsynaptic density protein 95
tPA: tissue plasminogen activator
